# Demethylation and Acetylation Modification of Alkali Lignin and Their Potential Applications in Sunscreen

**DOI:** 10.3390/polym18020286

**Published:** 2026-01-21

**Authors:** Jianan Hu, Yunni Zhan, Xuelian Zhou

**Affiliations:** 1 Jiangsu Co-Innovation Center of Efficient Processing and Utilization of Forest Resources, International Innovation Center for Forest Chemicals and Materials, Nanjing Forestry University, Nanjing 210037, China; 2 National Key Laboratory for Development and Utilization of Forest Food Resources, Institute of Chemical Industry of Forest Products, Chinese Academy of Forestry, Nanjing 210042, China

**Keywords:** alkali lignin, demethylation, acetylation, ultraviolet absorption, sunscreen

## Abstract

In order to improve the utilization of alkali lignin (AL) as an effective component for ultraviolet (UV) shielding, demethylation and acetylation modification were carried out to improve the UV absorption performance of lignin. Then, lignin-based sunscreens were successfully prepared by mixing the modified lignin and commercial cream without UV shielding ingredients. The modified alkali lignin was comprehensively characterized in terms of its molecular weight, functional groups and structural properties by GPC, UV spectroscopy and ^31^P NMR. The results showed that the Mw of all three lignin feedstocks (AL, AL_MeOH_ and AL_Acetone_) was decreased with prolonged demethylation time. Compared to the original feedstock, demethylated AL had a darker color and improved UV absorption performance due to the increased phenolic hydroxyl content (approximately 4.35 mmol/g). ^31^P-NMR spectra showed that the guaiacyl phenolic hydroxyl content decreased rapidly after acetylation, causing the sample color to become lighter. Among all lignin-based sunscreens, DAL_Acetone_ achieved the highest SPF value of 11.23, a 69.4% increase over its pre-reaction level and a 7.58-fold enhancement compared to the original lignin. In summary, this study opens a promising avenue for repurposing industrial lignin as a sustainable biomaterial in high-value sectors like UV-blocking agents and cosmetic formulations.

## 1. Introduction

Lignin is a complex organic compound widely found in plant cell walls, primarily composed of phenylpropane monomers. It serves as one of the main components of plant cell walls, accounting for over 50% of the total weight of plant cell walls. Lignin is the most abundant polyphenolic substance in nature [1,2]. Its primary production processes include wood processing, papermaking, and cellulose extraction, often recovered as low-cost waste from the pulp and paper industry and biorefineries. However, it has not yet been fully or efficiently utilized. Currently, there is growing emphasis on the development and utilization of industrial lignin, and the transformation and application of lignin and its derivatives have become a major research focus [3]. Industrial lignin mainly originates from black liquor produced in traditional pulp and papermaking processes and residues from biomass refining, with an annual output of approximately 50 million tons [4]. In wood processing, lignin can be obtained by treating sawdust. However, over 95% of industrial lignin is only used as fuel, resulting in low utilization efficiency. With the increasing awareness of environmental protection and sustainable development, the reuse of lignin has become increasingly important [5].

In recent years, the excellent ultraviolet (UV) radiation resistance of lignin has garnered significant attention from researchers. Lignin is a natural, broad-spectrum sunscreen active substance. Its effectiveness as an alternative sunscreen ingredient primarily depends on its source, composition, and specific structure. Provided that appropriate treatment methods are employed to directionally adjust, control, and structurally modify lignin, it holds great application potential [6,7,8]. It can be developed into a natural polymeric sunscreen agent for sun protection and skincare [9]. With the growing awareness of environmental protection, natural and eco-friendly attributes are considered the primary reasons for attracting consumers to purchase lignin-based sunscreens [10,11]. Li et al. designed five different solvent systems to extract lignin samples from sugarcane bagasse. The resulting lignin sample revealed the lowest total color difference and highest sun protection factor (SPF of 3.99) [12]. The SPF of commercial facial creams significantly increased after the addition of 5 wt% lignin sample, and the lignin-modified creams exhibited excellent broad-spectrum UV absorbency. The effects of different molecular weights of lignin on the color and UV-protecting property of lignin-based sunscreens were also investigated [13]. The lower the molecular weight of lignin, the higher the SPF of the lignin-based sunscreen (SPF of 7.14). Additionally, adding TiO_2_ could efficiently mitigate the dark color of lignin-based sunscreens due to its prominent covering power. However, lignin currently does not fully meet the requirements for commercial use, mainly because the sun protection factor (SPF) value of untreated lignin is insufficient to satisfy the demands of most users. At present, the main issue with lignin as a sunscreen active substance is that its structure is severely disrupted during the pulping process [14]. This process generates a large number of chromophores and condensed structures, which darken the color of lignin. As a result, sunscreen products prepared in this way cannot achieve the pure white appearance of most commercially available sunscreen products, failing to meet consumer expectations. Another issue is that the UV-blocking performance of untreated lignin remains relatively low and still falls short of current commercial standards [15]. Recent work reported that organic solvent fractionation could effectively improve the UV absorption performance and reduce the apparent color of lignin. Due to the increased phenolic hydroxyl content of the soluble lignin fraction, the lignin-based sunscreen exhibited a higher sun protection factor [16]. Therefore, exploring methods to prepare lignin with excellent UV-blocking properties and a lighter appearance, as well as developing high-performance UV-shielding materials, is a crucial step in promoting innovation and progress in novel lignin-based functional materials [17]. This has significant implications for the efficient utilization and development of lignin.

The hydroquinone groups in lignin can convert between hydroquinone and quinone forms after absorbing ultraviolet energy, forming conjugated structures that endow this biomass material with excellent UV-shielding properties. This process primarily functions through structural interconversion between the ground state and excited state: molecules in the ground state absorb energy from UV radiation and transition to an excited state; however, the excited state, having absorbed energy, is unstable and reverts to the ground state while releasing excess energy [18,19]. Typically, the demethylation reaction of lignin is utilized, in which methoxy groups on the benzene rings of lignin are oxidized by oxidants into hydroxyl groups, forming phenolic hydroxyl structures. A practical challenge in this process is the precise control of the degree of methoxy oxidation. Strong oxidants, such as potassium permanganate, can directly disrupt the benzene rings, breaking them down into low molecular weight acids. Therefore, a mild oxidant is required to selectively cleave the ether bonds between two propylphenyl units and generate more phenolic hydroxyl groups along with hydroquinone groups. Hydrogen peroxide serves as an excellent oxidant in this context. It reacts with ferrous ions to produce hydroxyl radicals, which can then oxidize lignin via the Fenton reaction, thereby increasing the phenolic hydroxyl functional group content in lignin [20]. During this reaction process, the degree of lignin oxidation is controlled by adjusting the reaction time.

Herein, alkali lignin was fractionated using organic solvents, then chemically modified through demethylation and acetylation treatments to enhance its phenolic hydroxyl group content and ultraviolet absorption performance. The modified lignin was then uniformly mixed with a commercial cream base to prepare lignin-based sunscreen samples. The sun protection factor (SPF) of the samples was tested to explore the application prospects of lignin in the cosmetics field.

## 2. Materials and Methods

### 2.1. Materials and Chemicals

Alkali lignin (AL) was purchased from Sigma-Aldrich (Shanghai, China). Acetic anhydride, pyridine, and potassium bromide (KBr) were purchased from Sinopharm Chemical Reagent Co., Ltd. (Shanghai, China); Acetone and methanol (MeOH) were ordered from Nanjing Chemical Reagent Co., Ltd. (Nanjing, China); Sodium hydroxide (NaOH) and hydrochloric acid (HCl) were purchased from Nanjing Chemical Reagent Co., Ltd. (Nanjing, China); Tetrahydrofuran (THF), hydrogen peroxide (30 wt%, AR) and ferrous chloride (FeCl_2_) were purchased from Aladdin Chemical Reagent Co., Ltd. (Shanghai, China). NIVEA Men Moisturizing Cream (NIVEA cream, NIVEA Shanghai Co., Ltd., Shanghai, China) was purchased from NIVEA Tmall Mall. All the above reagents were of analytical grade and used without further purification.

### 2.2. Organic Solvent Treatment

A total of 1 g of AL was slowly added to 200 mL of a 75 wt% organic solvent/water mixed solution. After stirring for 30 min at room temperature, the solution was vacuum filtered using filter paper. The obtained soluble fraction was then subjected to rotary evaporation under vacuum to remove the organic solvent, followed by freeze-drying to obtain the dried sample. For simplicity, the soluble fractions derived from different organic solvent/water solutions are abbreviated as AL_MeOH_ and AL_Acetone_.

### 2.3. Demethylation of Lignin

Demethylation of lignin was achieved through the Fenton reaction. To be specific, 1 g of lignin sample was dissolved in 200 mL of acetone/water (7/3, *v*/*v*) solvent, followed by adding 4 mg of FeCl_2_ as a catalyst and 50 mL of hydrogen peroxide aqueous solution (30 wt%) as the oxidant. The reaction was conducted at room temperature for 3–5 h. The mixture was finally filtered and freeze-dried to obtain the demethylated AL, named DAL.

### 2.4. Acetylation of Lignin

In total, 0.1 g of the sample was dispersed in pyridine with the addition of 1 mL of acetic anhydride. After the reaction proceeded for 72 h, the mixture was gradually dripped into 20 mL of ice-cold water (pH < 2, adjusted with HCl) under continuous stirring. The precipitate was washed with deionized water and then freeze-dried to obtain acetylated lignin. For simplicity, the demethylated and acetylated AL was named ADAL.

### 2.5. Gel Permeation Chromatography (GPC) Analysis

The number of average molecular weight (M_n_) and weight-average molecular weight (M_w_) of lignin samples were determined by Agilent GPC (LD-50, Agilent Technologies Inc., Santa Clara, CA, USA) equipped with KF-803 columns and a refractive index detector (RID). Chromatographically pure tetrahydrofuran was used as the mobile phase. Polystyrene was used to calibrate the test standards within the range of 2–100 k. The volume of injection was 10 μL in each run, and the column temperature was maintained at 30 ± 0.1 °C.

### 2.6. FTIR Analysis

Fourier Transform Infrared Spectroscopy (FT-IR, Nicolet 6700, Thermo Scientific, Waltham, MA, USA) was used to determine the infrared spectra of the raw lignin and the lignin after demethylation and acetylation. The measurement range was 4000–400 cm^−1^ with 32 scans. The potassium bromide pellet method was employed for testing. Lignin samples were dried in an oven at 60 °C for 3 h. Subsequently, 200 mg of KBr and 2 mg of the sample were thoroughly mixed and ground finely in an agate mortar. The mixture was then pressed into uniform thin pellets using a pellet press for measurement.

### 2.7. NMR Analysis

^1^H-NMR and ^31^P-NMR were used to investigate the structure of the functionalized modified lignin as previously reported [21]. Prior to testing, the lignin sample was dried in a constant temperature oven at 60 °C for 12 h.

^1^H-NMR: Approximately 40 mg of the dried acetylated lignin sample was dissolved in 0.6 mL of deuterated dimethyl sulfoxide (DMSO-d6). Using tetramethylsilane (TMS) as the internal standard, the sample was analyzed with a 600 MHz fully digital superconducting nuclear magnetic resonance spectrometer (Bruker Co., Ettlingen, Germany). The test temperature was 25 °C, with a scanning duration of 9 h. TopSpin 4.4.0 software was used to calculate the peak area integrals for the functional groups.

^31^P-NMR: 25 mg of the lignin sample was dissolved in 400 μL of a mixed solvent of anhydrous pyridine/deuterated chloroform (CDCl_3_) (1.6:1, *v*:*v*). Then, 100 μL of the internal standard cyclohexanol (4.0 mg/mL in pyridine/CDCl3 solution) and 50 μL of the relaxation reagent chromium(III) acetylacetonate (3.6 mg/mL in pyridine/CDCl_3_ solution) were added to the mixture. After thorough mixing, 75 μL of the phosphorylation reagent 2-chloro-4,4,5,5-tetramethyl-1,3,2-dioxaphospholane (TMDP) was added and mixed well before analysis.

### 2.8. Evaluation of Color Change

The color reference values L*, a*, b* of lignin samples were measured using xrite Xact spectrodensitometer (Ashley color technology Co., Ltd., Shanghai, China). Before testing, automatic color calibration was performed. Lignin samples were placed in colorless transparent bags and spread into a dense, opaque layer of a certain aera for testing. The color parameters L*, a*, b* (L* = brightness, a* = red/green and b* = yellow/blue) of the sample were recorded, and each sample was tested for five times to determine the average value.

### 2.9. UV-Light Spectrum Analysis

The lignin samples were dissolved in a 1 mol/L NaOH solution, and their absorbance was then measured using a UV-Vis spectrophotometer (UV-1780, Shimadzu Corp., Kyoto, Japan). Prior to testing, an aqueous NaOH solution was scanned at the same wavelength to establish the baseline.

### 2.10. Preparation and Characterization of Lignin-Based Sunscreen

Aiming at the application of the target lignin in natural sunscreen agents, a lignin-based sunscreen formulation was prepared by simulating the traditional method of blending ultraviolet-shielding active substances with moisturizing cream. Specifically, 0.1 g of lignin and 1.9 g of commercial cream free of ultraviolet-blocking ingredients were mixed at room temperature at 750 rpm for 24 h to ensure uniform dispersion of the lignin in the cream, yielding a sunscreen containing 5 wt% lignin. Then, 3 M perforated tape was applied to a clean 2 mm thick quartz glass slide to simulate human skin. After mixing, 0.4 g lignin sunscreen was transferred evenly to the surface of the glass slide with tape, and the ultraviolet transmittance was measured with an ultraviolet spectrophotometer. Transmittance measurement per 1 nm was gathered in the wavelength range from UVB (290–320 nm) to UVA (320–400 nm). The SPF value was calculated by the following formula:

$$SPF=\sum_{290}^{400}E_{\lambda }S_{\lambda }/\sum_{290}^{400}E_{\lambda }S_{\lambda }T_{\lambda }$$

where E_λ_ = erythema spectral effectiveness, i.e., the effect of sunlight at the wavelength to cause human erythema, S_λ_ = Solar spectral effectiveness, the energy intensity of sunlight irradiating the ground at the wavelength T_λ_ = Spectral transmittance of the sample.

## 3. Results

### 3.1. Molecular Weight of Lignin After Methylation Reaction

The Fenton reaction is a redox reaction that involves adding hydrogen peroxide and iron ions (usually Fe^2+^) to produce radical hydroxyl groups (·OH) under acidic conditions. These radicals have strong oxidizing properties and can react with organic matter such as lignin, as shown in reaction Equation (1). However, if the amount of Fe^2+^ is too low, it will not cause the consumption of hydrogen peroxide. Without the consumption of hydrogen peroxide, the Fenton reaction does not occur, and no hydroxyl radicals are generated to oxidize lignin. Therefore, it is necessary to ensure that the amount of Fe^2+^ is sufficient.

(1) $${\mathrm{Fe}}^{2+}+{\;H}_{2}O_{2}\to {\mathrm{Fe}}^{3+}+\mathrm{OH}+{\mathrm{OH}}^{-}$$

**Figure 1:**
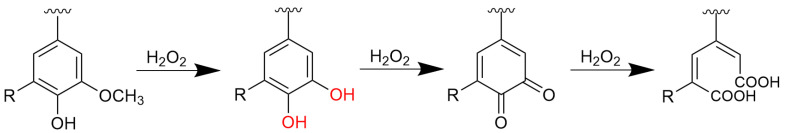
The process of lignin changes in the Fenton reaction.

But it should be noted that the Fenton reaction is a non-selective oxidation reaction. In application, appropriate treatment methods need to be selected according to specific circumstances to achieve the best treatment effect. When the concentration of Fe^2+^ in the solution is maintained at a sufficient level, the reaction can reach the highest yield. Furthermore, the reaction intermediates are stable under these conditions. The process diagram of hydrogen peroxide oxidizing lignin is shown in Figure 1.

The Fenton reaction cleaves the molecular chains of lignin, thereby reducing its weight-average molecular weight (M_w_), while also generating low-molecular-weight organic acids and other small-molecule compounds. Due to the structural complexity of lignin, the specific changes in its M_w_ depend on factors such as reaction conditions, duration, and the initial lignin structure. As shown in Table 1, the M_w_ of lignin initially increased slightly and then decreased with prolonged demethylation time. When the raw lignin reacted for 3–4 h, the oxidation of methoxy groups to phenolic hydroxyl groups enhances its hydrogen-bonding capacity. During the subsequent reassembly process, the polymerization degree of lignin increased compared to that before the reaction, leading to a rise in M_w_ within this period. In general, however, the Fenton reaction tended to depolymerize lignin. After 5 h of reaction, the M_w_ of all three lignin feedstocks (AL, AL_MeOH_ and AL_Acetone_) decreased, confirming that prolonged oxidation induced ring-opening reactions in lignin, resulting in a reduction of molecular weight.

### 3.2. FTIR Analysis

FTIR analysis revealed distinct characteristic bands in the samples corresponding to lignin aromatic rings (1422 cm^−1^, 1510 cm^−1^, 1596 cm^−1^), including the peak at 1510 cm^−1^ attributed to the skeletal vibration of the phenyl-propane structure [22]. These observations indicate that the lignin structure remained largely intact during demethylation. Specifically, the peak at 3400 cm^−1^ is assigned to hydroxyl group stretching, while the bands at 1457 cm^−1^ and 1364 cm^−1^ originate from C–H stretching in methoxy groups and acetyl groups [23,24], respectively. The secondary hydroxyl absorption at 1212 cm^−1^ is considered responsible for ultraviolet absorption. Further peaks were identified at 2929 cm^−1^ (skeletal vibration of methyl/methylene carbon chains), 1717 cm^−1^ (carbonyl group vibration), and 1263/1244 cm^−1^ (C–O vibration in phenolic hydroxyl groups). The signal at 1209 cm^−1^ is attributed to combined C–C, C–O, and C=O vibrations within the aromatic region, whereas the absorptions at 1590/1510 cm^−1^ and 1424 cm^−1^ correspond to aromatic skeletal vibrations [25,26]. The relatively weak peak at 1120 cm^−1^ suggests only trace amounts of syringyl structures are present in the lignin.

**Figure 2:**
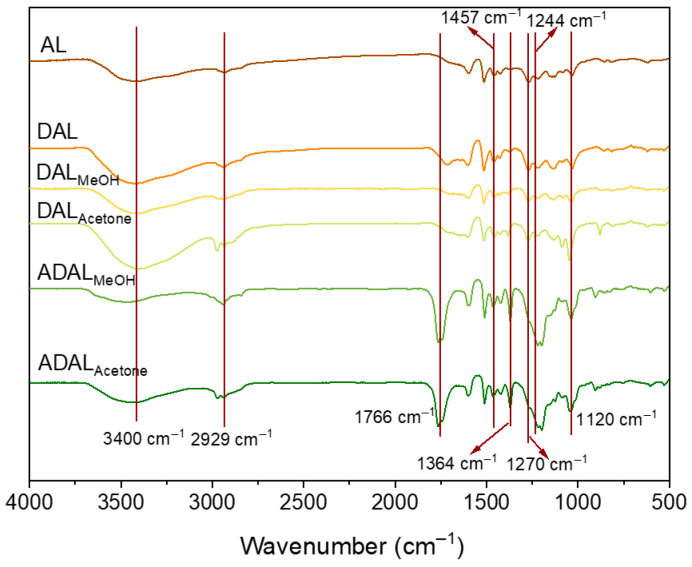
FTIR of lignin samples after demethylation and acetylation.

The intensity of the characteristic C=C bond absorption peaks of the benzene ring (at 1600 and 1510 cm^−1^) decreased significantly, demonstrating that excessive oxidation for over 5 h induced further reactions of phenolic hydroxyl groups, leading to the formation of unwanted by-products. FTIR analysis further reveals that the Fe^2+^/H_2_O_2_ system effectively modulates the oxidation degree of lignin. As illustrated in Figure 2, the hydroxyl stretching vibration at 3400 cm^−1^ broadened and intensified, whereas the ether bond stretching vibration associated with methoxy groups at 1270 cm^−1^ weakened. These changes confirm the oxidation of methoxy groups and the generation of catechol structures in the demethylated samples, DAL_MeOH_ and DAL_Acetone_. Following acetylation, the hydroxyl stretching peak at 3400 cm^−1^ in ADAL_MeOH_ and ADAL_Acetone_ lignin samples notably diminished. Simultaneously, a distinct C=O stretching vibration emerged at 1766 cm^−1^, verifying the successful introduction of acetyl groups. Moreover, the appearance of a marked peak at 1200 cm^−1^ indicates that a portion of the catechol structures was oxidized to quinones [27].

### 3.3. Functional Group Analysis

To quantitatively determine the exact content of phenolic hydroxyl and carboxyl groups in lignin samples, ^1^H-NMR and ^31^P-NMR were used to analyze the functional group content of lignin samples.

**Figure 3:**
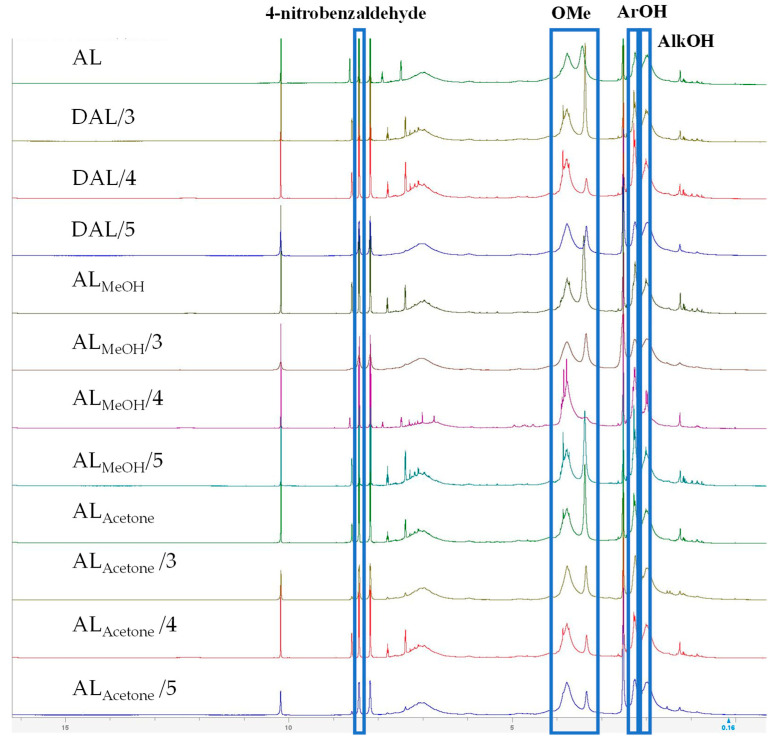
Functional groups of different lignin samples determined by ^1^H-NMR spectra.

Figure 3 shows the ^1^H-NMR spectra of different lignin samples; the proton signals of methoxy groups, phenolic hydroxyl group, and aliphatic hydroxyl group were located at 3.10–4.10, 2.17–2.50, and 1.70–2.17 ppm, respectively [28]. As shown in Table 2, the methoxy group content decreased significantly during demethylation, with the rate of decrease slowing after 4 h. Concurrently, the phenolic hydroxyl content exhibited an initial increase followed by a decline as the treatment progressed, peaking at the 4-h mark. Among the samples, DAL_MeOH_ reacted for 4 h showed the highest phenolic hydroxyl concentration (4.47 mmol/g), representing a 73.26% increase over the raw AL value of 2.58 mmol/g. Furthermore, the aliphatic hydroxyl group content in all three lignin samples rose continuously with prolonged treatment. Notably, the aliphatic hydroxyl content of the DAL_Acetone_ sample increased markedly from 3.66 to 4.44 mmol/g, providing further evidence that the aliphatic side chains continue to undergo demethylation reactions when the reaction exceeds 4 h.

**Figure 4:**
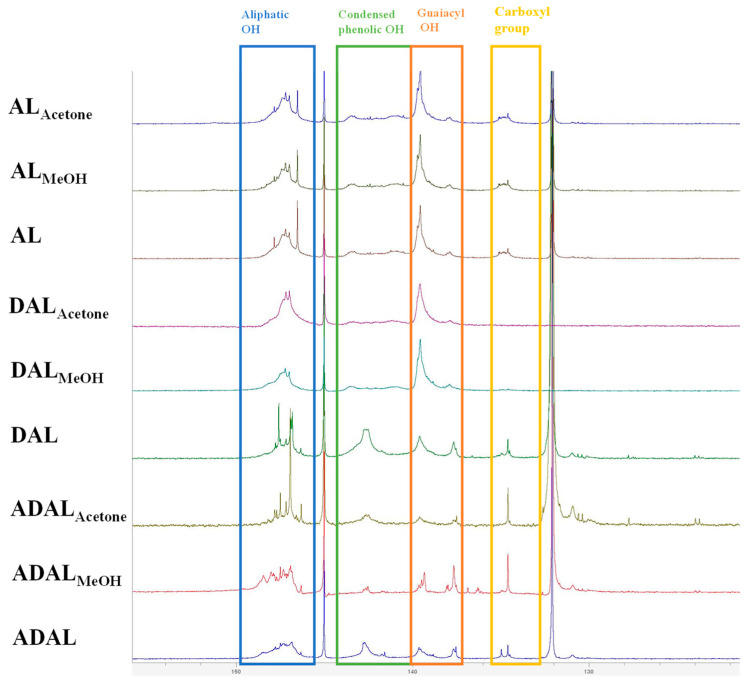
Functional groups of different lignin samples determined by ^31^P-NMR spectra.

^31^P-NMR spectroscopy offers a convenient, direct, and accurate method for quantifying all active hydroxyl groups in lignin, including phenolic, aliphatic, and carboxyl groups [29]. Figure 4 showed the ^31^P-NMR spectra of different lignin samples; the aliphatic -OH, condensed phenolic -OH, and guaiacyl -OH were located at 145.6–149.0, 140.4–144.4, and 137.6–140.4 ppm, respectively [30]. As shown in Table 3, the demethylation reaction resulted in minimal changes to the aliphatic hydroxyl and condensed phenolic hydroxyl contents across the three lignin samples. The most notable increase was observed in guaiacyl phenolic hydroxyl groups, which constitute the target functional group in this study. This preference stems from the ability of guaiacyl phenolic hydroxyl groups to effectively produce a conjugation effect. Moreover, upon absorbing ultraviolet energy, these hydroxyl groups on the benzene ring can undergo tautomerism with ketone carbonyl structures, thereby enhancing the UV-shielding performance of the lignin. In contrast, acetylated demethylated lignin samples showed a pronounced reduction in both condensed phenolic hydroxyl and guaiacyl phenolic hydroxyl groups, particularly the latter. This trend aligns with observations from infrared spectroscopy, indicating a high acetylation degree of 50–74%. Such a decrease is attributed to the substitution of hydroxyl groups on the aromatic ring by acetyl groups during the acetylation reaction.

### 3.4. Chromaticity Analysis

**Figure 5:**
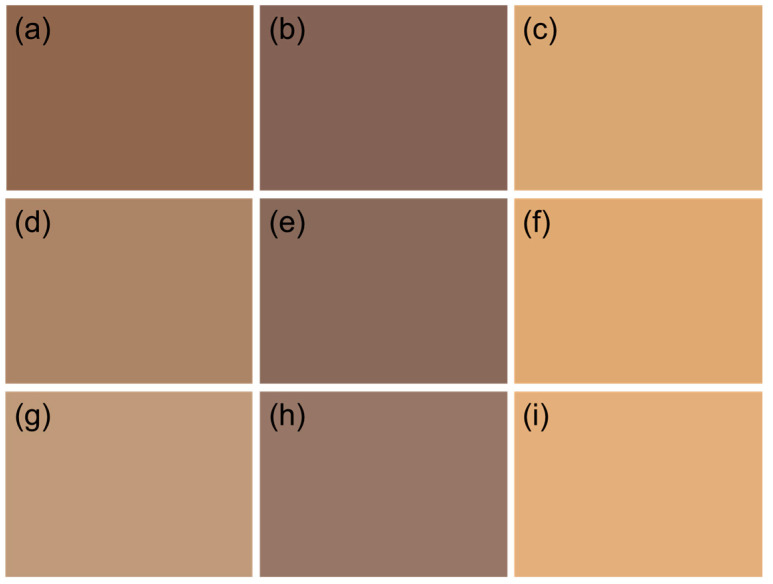
Lab color comparison of lignin fractions of (**a**) AL, (**b**) DAL, (**c**) ADAL, (**d**) AL_MeOH_, (**e**) DAL_MeOH_, (**f**) ADAL_MeOH_, (**g**) AL_Acetone_, (**h**) DAL_Acetone_ and (**i**) ADAL_Acetone_.

As shown in Figure 5, the apparent color of DAL, DAL_MeOH_ and DAL_Acetone_ darkened considerably after demethylation. This color change was attributed to the oxidation of most methoxy groups in lignin to phenolic hydroxyl groups during the reaction. Phenolic hydroxyl groups acted as chromophoric functionalities in lignin and could undergo interconversion with ketone structures, promoting condensation reactions and consequently darkening the lignin color [31]. Furthermore, the enhanced phenolic hydroxyl content strengthened the hydrogen-bonding capacity of lignin. This resulted in the formation of larger microsphere structures during reassembly and a higher overall bulk density of the sample compared with untreated lignin. Therefore, the increase in chromophores within lignin, together with elevated bulk density, raised the macroscopic concentration of chromophoric groups, which significantly deepened the color of the lignin. Even for the relatively light-colored AL_Acetone_, the L value of DAL_Acetone_ decreased by 13.2% after the reaction in Table 4. The reduction in L value of AL_MeOH_ was significantly higher than others, dropping by 19.3%. In contrast, the original AL showed only a 4.8% decrease in L value, which could be attributed to its initially low lightness value. The less pronounced decline in L value for DAL compared to the other samples was due to the fact that AL itself was already dark in color and did not undergo organic solvent fractionation.

The appearance of the lignin samples after acetylation showed a noticeable lightening in color, tending toward a light brown overall and becoming closer to human skin color. It could be observed that acetylation significantly enhanced the brightness value of lignin, as shown in Table 4. Among them, ADAL exhibited the greatest increase in brightness compared to DAL, with an improvement of 61%, which was attributed to DAL having the lowest L value. In contrast, ADAL_MeOH_ and ADAL_Acetone_ showed increases of 54.9% and 42.9%, respectively, compared to their demethylated counterparts. Although ADAL_Acetone_ exhibited the smallest percentage increase among the three samples, its brightness value was the highest. The lightening effect of acetylation on lignin color occurred because acetyl groups were introduced into the benzene ring of lignin, replacing the phenolic hydroxyl functional groups, which altered its molecular and electronic structure, leading to changes in its absorption spectrum and difference in color appearance.

### 3.5. UV Performance Analysis

**Figure 6:**
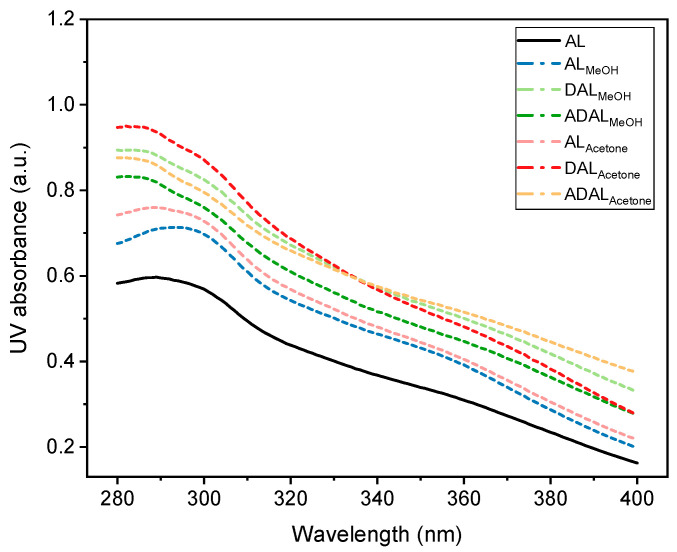
UV absorbance of different lignin after demethylation and acetylation at the same concentration.

The ultraviolet absorption characteristics of lignin vary depending on its molecular structure and chemical composition, leading to differences in absorption rates and wavelengths among different lignin types [32,33]. Typically, lignin exhibited strong ultraviolet absorption, with characteristic peaks appearing between 200 and 400 nm. The position and intensity of these peaks are influenced by the specific structural and chemical features of the lignin. A prominent absorption peak around 280 nm was commonly observed, which was mainly attributed to the aromatic rings and non-conjugated phenolic groups present in lignin. As shown in Figure 6, the demethylated samples DAL_MeOH_ and DAL_Acetone_ displayed the highest ultraviolet absorption rates at the same solution concentration compared to other samples. This enhancement was due to the increased phenolic hydroxyl content resulting from the demethylation reaction. The conjugation between phenolic hydroxyl groups and benzene rings, together with electron transitions following photon absorption, contributed to the improved ultraviolet absorption capacity of these lignin samples [34]. However, this increased absorption was accompanied by a tendency toward darker coloration in the treated lignin. In comparison, acetylated lignin samples generally exhibited a decrease in absorption intensity, but they showed a correspondingly lighter color. Moreover, while there was some reduction in the ultraviolet absorption rate, it still remained generally higher than that of the original lignin samples. Additionally, lignin samples fractionated with acetone/water mixed solvents typically demonstrated superior ultraviolet absorption capability compared to those treated with methanol-based solvents. Therefore, in practical applications, appropriate lignin treatment methods and reaction intensities should be selected based on specific requirements to achieve an optimal balance between ultraviolet absorption performance and coloration.

### 3.6. Sunscreen Performance

**Figure 7:**
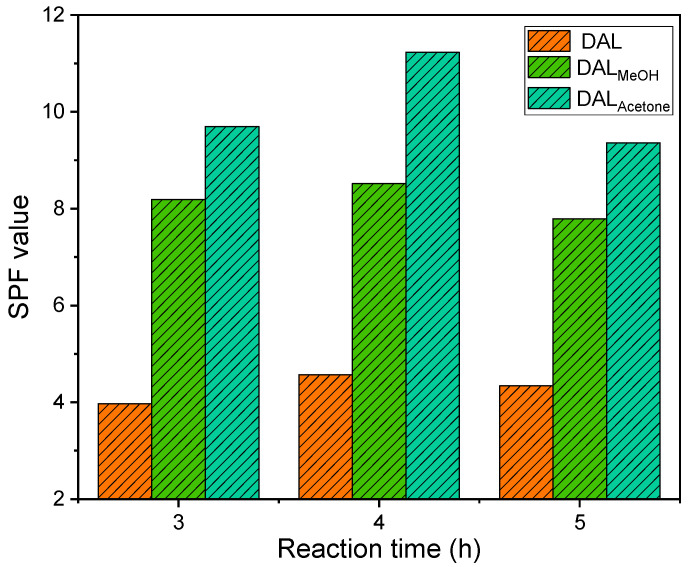
SPF values of different lignin samples after different demethylation reaction times.

As shown in Figure 7, the SPF values of lignin-based sunscreens increased significantly following the demethylation of lignin, which was attributed to the rise in phenolic hydroxyl groups. With extended reaction time, the SPF value initially increased and then declined, a trend consistent with changes in the phenolic hydroxyl content. Figure 5 further revealed that acetone-soluble lignin yielded a substantially higher SPF value after demethylation compared to the other two lignin feedstocks. The SPF of the lignin-based sunscreen peaked at 4 h of demethylation. Among the samples, the SPF order was DAL_Acetone_ > DAL_MeOH_ > DAL. Relative to its pre-reaction state, DAL_MeOH_ exhibited a 51.7% increase, while DAL_Acetone_ achieved the highest SPF value of 11.23, a 69.4% increase over its pre-reaction level and a 7.58-fold enhancement compared to the original lignin. At 3 h of reaction, incomplete oxidation left some methoxy groups unreacted, limiting phenolic hydroxyl formation. Beyond 4 h, prolonged reaction led to over-oxidation, resulting in ring-opening of lignin benzene rings and disruption of the conjugation between hydroxyl groups and aromatic systems. This diminished the UV-shielding performance and correspondingly lowered the SPF value. Notably, the SPF values after 3 and 5 h of reaction did not differ significantly.

After the acetylation reaction, the SPF value of lignin decreased but remained higher overall than that of the original lignin. This was attributed to the substitution of phenolic hydroxyl groups by acetyl groups during the reaction. The loss of phenolic hydroxyls weakened the conjugation effect within the lignin structure, thereby reducing its SPF. However, the C=O bonds introduced by acetylation could also engage in conjugation with the benzene rings, which partially sustains the UV-shielding performance of the modified lignin. 

Table 5 lists the SPF values of different lignin-based sunscreens. For acetylated lignin-based sunscreens prepared via organic solvent fractionation, the reduction in SPF value remained below 10%. The SPF values followed the order ADAL_Acetone_ > ADAL_MeOH_ > ADAL. Relative to their respective pre-acetylation baselines, ADAL_MeOH_ and ADAL_Acetone_ showed decreases of 8.1% and 9.1%, respectively. In contrast, ADAL did not form a nanosphere structure after acetylation, resulting in a more pronounced decline in UV shielding performance, with a 24.1% drop compared to its pre-reaction state. These results demonstrate that acetylation did not substantially affect the SPF of lignin samples that have previously undergone both organic solvent fractionation and demethylation. In addition, the resultant composite sunscreen displayed a closer or higher SPF value than those reported using other methods (e.g., lignin-modified cream, SPF 4.0; Eucalyptus lignin nanoparticles, SPF 7.0; lignosulfonate-based sunscreen, SPF 4.8–13.0) in the literature [12,35,36]. Although the SPF still did not meet the minimum standards (SPF > 15) for commercial products, the entire lignin purification process was relatively environmentally friendly, and no chemical additives were added, which provided a convenient and green pathway for the value-added utilization of lignin.

## 4. Conclusions

In order to improve the UV protection capabilities of lignin, alkali lignin was separated by organic solvents (methanol and acetone) fractionation, and then modified through demethylation and acetylation for the use of sunscreens. Compared to the original lignin, characterization revealed that the phenolic hydroxyl content was highest after 4 h of demethylation, while acetylation reduced guaiacyl phenolic hydroxyls by approximately 70%. Among all lignin-based sunscreens, the demethylated AL (DAL_Acetone_) exhibited the highest sun protection factor of 11.23, a 7.58-fold enhancement over native AL. These findings highlight a viable route to transform industrial lignin into a sustainable, high-performance ingredient for UV protection and cosmetics.

## Figures and Tables

**Table 1:** Molecular weight of lignin after methylation reaction at different reaction times.

Sample/Reaction Time (h)	M_n_	M_w_	PDI
AL	1965	7792	3.965
DAL/3	2861	9825	3.434
DAL/4	2534	9084	3.585
DAL/5	2236	8052	3.601
AL_MeOH_	1433	2755	1.922
DAL_MeOH_/3	1422	2816	1.980
DAL_MeOH_/4	1386	2886	2.082
DAL_MeOH_/5	1453	2638	1.815
AL_Acetone_	2240	8608	3.843
DAL_Acetone_/3	2753	9631	3.498
DAL_Acetone_/4	2498	9254	3.705
DAL_Acetone_/5	2604	9030	3.467

**Table 2:** Determination of functional groups in different lignin by ^1^H NMR.

Sample/Reaction Time (h)	OMe (mmol/g)	ArOH (mmol/g)	AlkOH (mmol/g)
AL	8.73	2.58	3.48
DAL/3	8.06	3.02	3.49
DAL/4	6.61	4.27	3.62
DAL/5	6.68	2.96	3.97
AL_MeOH_	9.70	3.73	3.66
DAL_MeOH_/3	9.18	3.90	4.58
DAL_MeOH_/4	8.67	4.47	2.76
DAL_MeOH_/5	8.15	4.36	3.66
AL_Acetone_	8.38	3.49	3.66
DAL_Acetone_/3	8.15	4.01	4.33
DAL_Acetone_/4	7.31	4.02	4.44
DAL_Acetone_/5	6.76	3.24	4.25

**Table 3:** Phenolic and aliphatic hydroxyl contents of different lignins determined by ^31^P-NMR.

mmol/g	AL	AL-MeOH	AL-Acetone	DAL	DAL-MeOH	DAL-Acetone	ADAL	ADAL-MeOH	ADAL-Acetone
Aliphatic -OH	1.80	1.76	2.53	1.31	1.28	1.73	1.22	1.30	1.64
Condensed phenolic -OH	1.10	1.18	1.28	0.83	0.75	0.88	0.53	0.50	0.55
Guaiacyl -OH	1.54	1.94	1.61	1.65	2.27	2.22	0.78	0.62	0.49
Carboxyl group	0.33	0.43	0.25	0.22	0.15	0.13	0.35	0.39	0.37

**Table 4:** The chromaticity values of different lignin fractions after demethylation and acetylation.

Sample	L	a	b	Increasing Rate of L
AL	46.09	10.27	17.84	
DAL	43.88	8.66	10.89	−4.8
ADAL	70.65	10.02	30.82	61.0
AL_MeOH_	57.30	8.64	19.28	
DAL_MeOH_	46.24	6.79	11.11	−19.3
ADAL_MeOH_	71.62	10.63	33.12	54.9
AL_Acetone_	59.27	9.27	20.11	
DAL_Acetone_	51.47	7.56	11.84	−13.2
ADAL_Acetone_	73.57	10.52	31.27	42.9

**Table 5:** Comparison of SPF values of different lignin-based sunscreens in this work.

Sample	SPF Value	Increased Proportion
AL	1.48	
DAL	4.57	208.78%
ADAL	3.47	−24.07%
AL_MeOH_	5.63	
DAL_MeOH_	8.52	51.33%
ADAL_MeOH_	7.83	−8.1%
AL_Acetone_	6.63	
DAL_Acetone_	11.23	69.4%
ADAL_Acetone_	10.20	−9.17%

## Data Availability

The original contributions presented in this study are included in the article. Further inquiries can be directed to the corresponding author.
